# Hyper-exponential growth in an epidemic model: some explicit solutions and their significance

**DOI:** 10.1007/s00285-026-02437-8

**Published:** 2026-07-17

**Authors:** John M. Halley

**Affiliations:** Laboratory of Ecology, Department of Biological Applications and Technology, University of Ioannina, 45110 Ioannina, Greece

**Keywords:** Hyper-exponential growth, Super-exponential growth, Mutation, SIR model, Transmission coefficient, Finite-time singularity, 91-10

## Abstract

The COVID-19 pandemic highlighted the ability of epidemics to evolve through the emergence of successive strains of greater infectiousness, which prompted the insight that hyper-exponential growth (HEG) can arise in the development of an epidemic. The phenomenon of HEG has intrigued many researchers, because of some radical differences from exponential growth such as the finite-time singularity. However, the actual mechanism of HEG was usually hidden or needed to be added phenomenologically. Thus, there was little insight into how constraints would be triggered. In this study, we explore an SIR model with evolving parameters leading to a discrete sequence of variants. This allows us to consider the HEG phenomenon in greater depth and with greater mathematical rigour. The model yields a mechanistic description of what happens at the collapse of HEG. The model also yields closed expressions for important features, such as the critical time to the singularity, in terms of basic demographic parameters. Our analysis flags a few important issues needing further research, such as the stochastic character of the emergence of variants. Greater understanding of the HEG process will yield dividends in other fields, since modern societies exhibit HEG at several levels, such as human population growth, economic indicators and technological innovation. For this reason, more research on HEG remains imperative, especially on HEG in the presence of limited resources.

**Supplementary Information:**

The online version contains supplementary material available at 10.1007/s00285-026-02437-8.

## Introduction

**Fig. 1:**
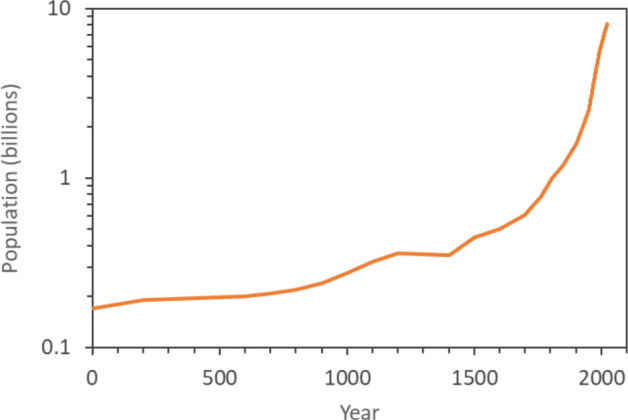
Human population of the Earth (in billions) between AD 0–2020, drawn with a log vertical scale. Growth rate up to 1500 could plausibly be considered as exponential but must be considered as hyper-exponential from 1500 until the late 1960s

Hyper-exponential growth (HEG) can be understood as accelerating exponential growth where growth rate itself is increasing. The difference between HEG and the much-studied exponential growth is illustrated on a graph with a logarithmic vertical axis. HEG retains an upward curvature, while exponential growth is linear. The global human population through history (Worldometers 2024) is a case in point for HEG (Fig. 1). For most of history the human growth rate appears to be accelerating. This means that the doubling-time for population was getting shorter as population rose. Supposing the human race began with a single couple, the human population doubled 32 times since our appearance (Hern 1999). Malthus had claimed that human population growing exponentially would always outstrip any growth of resources since resources could only increase linearly at best (Malthus 1798) and that limiting factors would act though the agencies of hunger and disease so as to limit population growth. However, human population grew more than exponentially. The many disasters occurring throughout human history did not stop this growth. Instead, improving technology, sanitation, agriculture and medicine enabled humans to circumvent these limiting factors. Then, between 1965 and 1970, a slowing of population growth occurred (Cohen 1995). Instead of negative forces of hunger and disease, this “demographic transition” has been explained by growing prosperity and education leading to more selective fertility and recently, declining birthrates in developed countries (Aitken 2024). The global human population, prior to 1965, is thus the paramount example of HEG.

Biological models that predict HEG are, however, rare. In population modelling, the basic dynamics is usually seen through the lens of the modified Malthusian equation for population size, *N*:

1 $$ \frac{1}{N}\frac{dN}{{dt}} \approx rN^{\alpha } $$

Here, the LHS is the per-capita growth per unit time and the term *rN*^*α*^ can be thought of as the excess births per unit of time and *r* the growth rate, assumed non-negative. Small ecological populations are usually modelled by this equation (or a stochastic equivalent) with *α* = 0 leading to exponential growth. Exponential growth in ecology has been likened to inertial motion in celestial mechanics (Ginzburg 1987; Turchin 2001): Just as a body remains in a state of uniform motion unless disturbed, so an ecological population remains in a state of exponential growth, this is also called the Malthusian law (Witting 1997). By contrast, if *α* < 0, the equation describes a (“density-dependent”) population with decelerating growth that eventually arrives at an equilibrium. The remaining possibility, a positive *α*, describes population growth when growth accelerates with *N*, leading to runaway HEG. The solution for Eq. (1) has been shown to exhibit power-law growth leading to a model of infinite population in a finite time (Cohen 1995; Johansen and Sornette 2001). This solution was discussed for the human population by von Foerster et al. who announced “Doomsday: Friday, 13 November, A.D. 2026” (von Foerster et al. 1960). This means that the model implied by Eq. (1) must break down or the system undergoes a regime shift (Kaack and Katul 2013; Parolari et al. 2015). Variants of this model, discussed at length in the context of human systems, are urban city sizes measured by their population (Bettencourt et al. 2007). In such an example, a modified von Bertalanffy equation results but with anabolism that grows faster than linear and a catabolism function that grows linearly.

Returning to Eq. (1), a more serious problem with the *α* > 0 version is finding a plausible biological mechanism. While it is easy to find factors that force *α* < 0 (e.g. resource depletion, intraspecific competition), plausible mechanisms that yield sustained *α* > 0 have proved more elusive. This elusiveness is most evident in ontogenetic growth models summarized by a von Bertalanffy type equation, where both anabolism and catabolism grow sub-linearly in size. Most of the identified mechanisms of hyper-exponential growth in biology appear to occur when organisms evolve (Witting 1997; Hern 1999; Halley et al. 2021), which is the main focus here. It may be shown (Witting 2000) that if one takes account of Fisher’s fundamental theorem of natural selection (“The rate of increase in fitness of any organism at any time is equal to its genetic variance in fitness at that time”), then one must replace *r* in Eq. (1) by *r* + *σ*^2^*t* and thus HEG behavior develops automatically even when *α* = 0. In this case,

$$ N \approx N_{0} exp\left[ {rt + \sigma^{2} t^{2} /2} \right]. $$

This happens because faster growth rates appear by chance, which are “selected” by virtue of simply growing faster.

Since evolution plays an important role in the emergence of infectious diseases (Antia et al. 2003; Woolhouse et al. 2005), we might expect HEG to arise is in the growth of epidemics (Halley et al. 2021). The COVID-19 pandemic involved the emergence of successive variants of SARS-CoV-2 with ever higher transmission potential, or higher ability to infect previously low-risk groups, or better resistance to neutralizing effects and vaccines, though some aspects of this picture have been contested (van Dorp et al. 2020). This was often (but not always) accompanied by milder symptoms, which might be thought as counter-intuitive in the context of evolutionary theory, where higher infectiousness tends to be accompanied by greater virulence. However, this is not always the case, as shown by Leggett et al. (2017). As well as HEG, Schwarzendahl et al. suggested that infection revivals could occur with mutations driving single waves or wave trains after a long period of decline of infectious individuals (Schwarzendahl et al. 2022). Accelerating rates can also appear in the speed of the advance wave of invading organisms, with speeds increasing in later generations, both in spatial models (Cobben et al. 2017; Bovier and Hartung 2023) and in real populations (Fitzpatrick et al. 2010). Halley et al. (2021) showed that some features of COVID, such as the observed pattern of dominance of successive variants, could be predicted by an SEIR model with evolving variants. However, a pronounced HEG character was not apparent in the COVID pandemic, since the growth of the pandemic was significantly affected by the various human countermeasures (Maier and Brockmann 2020).

The approach here generalizes and develops that of the earlier study (Halley et al. 2021), deriving explicit solutions for the conditions and time of emergence of HEG and approximations for wider conditions. This includes the hyper-exponential growth and collapse of an epidemic, using an SIR model with evolving parameters. This allows us to consider the HEG phenomenon in greater depth, and mathematical rigour, than previously seen. In the model, a cascade of variants each with successively higher infectiousness evolves. Every new strain grows faster to become the dominant strain, faster than the exponential growth of the existing strains. The mechanism of HEG, in this process, is transparent so that we can also observe the growth and collapse of HEG due to limited resources. This yields explicit solutions for the timing, conditions and exponent of the finite-time singularity, which in general depends on parameter values. We numerically solve the equations for the collapse of the epidemic and comment on the likely role of stochasticity. We also consider some implications of this model.

## The S.I.R. model with evolving variants

We use an approach similar to that in an earlier paper (Halley et al. 2021), with a system of equations with neither age-structure nor spatial-structure. We use the SIR model equations (Vynnycky and White 2010) with the possibility of mutation of the transmission coefficient. Specifically, we assume that initially susceptible individuals are infected by a single strain with transmission parameter *β*_0_. Mutations can change the transmission parameter, creating new variants, but other parameters do not change. For example, the recovery rate, *γ*, is assumed to be the same for all variants. Thus, variants are defined entirely by value of the transmission parameter. We assume that every mutation alters the transmission parameter by a fixed amount ± Δ*β* only. Thus, a new variant with increased transmission parameter (*β* → *β* + Δ*β*), appears with a small probability, *p*, per infected individual. While this is a small number (p≪1), nevertheless it is assumed large enough so that the number of successful mutations in an infected population of *I* will be approximately *pI*, without need for recourse to the discrete nature of infections. The mutation is assumed to occur “in flight” between the infectious and the infected person. The strains are labelled by index *m* = 0, 1, 2, …, *K*. The index here refers to the order of the strain. We can thus define the transmission coefficients as:

2 $$ \beta_{m} = \beta_{0} + m\Delta \beta ,\quad m \ge 0 $$

**Fig. 2:**
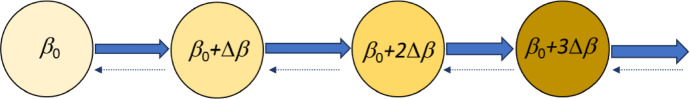
An SIR model for an epidemic with evolving between a discrete series of variants. Conceptual flow between variants is shown here. Flow towards less transmissive variants (dotted arrows) are ignored because they have small effect in an exponentially growing system

Successful variants (order-1, order-2, order-3, etc.), with transmission rates *β*_1_, *β*_2_*, β*_3_ and so on, lead to higher reproductive numbers *R*_0_^(1)^, *R*_0_^(2)^, *R*_0_^(3)^ …, respectively. Mutations “downward” (*β* → *β*-Δ*β*) are ignored as they have lower transmission coefficient. This is typical for RNA viruses that are causative agents of major diseases throughout human history including the COVID-19 pandemic. We can associate these additive increments with successively adapting traits. Our model follows each new strain that emerges from a significant mutation (Fig. 2).

In this model, each new variant, *m* > *0*, has its own subpopulation of infectious individuals (*I*_*m*_). Thus, if the total number of new strains is *K*, we have *K* + 1 infectious subpopulations including the original, leading to *K* + 3 equations in the system:

3 $$ \begin{aligned} & \frac{dS}{{dt}} = - S\sum\limits_{m = 0}^{K} {\frac{{\beta_{m} I_{m} }}{N}} \\ & \frac{{dI_{0} }}{dt} = + (1 - p)\left( {\frac{{S\beta_{0} }}{N}} \right)I_{0} - \gamma I_{0} \\ & \frac{{dI_{m} }}{dt} = + (1 - p)\left( {\frac{{S\beta_{m} }}{N}} \right)I_{m} - \gamma I_{m} + p\left( {\frac{{S\beta_{m - 1} }}{N}} \right)I_{m - 1} ,\quad \forall 1 \le m \le K \\ & \frac{dR}{{dt}} = + \gamma \sum\limits_{k = 0}^{K} {I_{m} } \\ \end{aligned} $$

The first equation is the equation for susceptibles. In this we see that the loss of susceptible individuals happens entirely through the conversion of susceptibles into infectious individuals, through transmission of the infection by any of the *K* + 1 variants. The second and third equations describes the number of individuals infected by the *k*th variant. This includes the number arriving because of infection of a susceptible individual by variant-*k* (1st term) and the number being lost from this class because of recovery or death (2nd term). Finally, for *m* > 0, an individual can enter this class, with probability *p*, through a mutation of the *k*-1th variant (3rd term). The last equation is the recovery of individuals from any of the *K* + 1 variants, which happens according to the same recovery rate, *γ*, for each variant. While all variants, according to Eq. (3), have nonzero population for any *t* > 0, we define a variant’s “appearance” as when its population surpasses at least one individual, “emergence” as when its growth rate is positive and its concentration humanly measurable, while a strain being “dominant” means that it has the largest subpopulation of the infectious classes.

## The rate equations and their solutions for early stages

If the epidemic is in a phase of “early” exponential growth, then *S*≈*N*. We can also use p≪1, so that that system of equations becomes:

4 $$ \begin{aligned} & \frac{dS}{{dt}} = - \sum\limits_{m = 0}^{K} {\beta_{m} I_{m} } \\ & \frac{{dI_{0} }}{dt} = (\beta_{0} - \gamma )I_{0} \\ & \frac{{dI_{m} }}{dt} = (\beta_{m} - \gamma )I_{m} + p\beta_{m - 1} I_{m - 1} ,\quad 1 \le m \le K \\ & \frac{dR}{{dt}} = + \gamma \sum\limits_{k = 0}^{K} {I_{k} } \\ \end{aligned} $$

The equations just for the variants, *I*_*m*_, can be rewritten in matrix notation, with the transition operator a lower bi-diagonal matrix of dimension(*K* + 1) × (*K* + 1).

5a $$ \frac{{d{\mathbf{I}}(t)}}{dt} = \left[ {\begin{array}{*{20}c} {a_{0} } & {} & {} & {} & {} \\ {b_{1} } & {a_{1} } & {} & {{\mathbf{\underline {0} }}} & {} \\ {} & {b_{2} } & {a_{2} } & {} & {} \\ {} & {} & {b_{3} } & {a_{3} } & {} \\ {{\mathbf{\underline {0} }}} & \ldots & \ldots & \ldots & \ldots \\ \end{array} } \right]\,\,{\mathbf{I}}(t)\quad {\mathrm{where}}\quad {\mathbf{I}}(t) = \left[ {\begin{array}{*{20}c} {I_{0} } \\ {I_{1} } \\ {I_{2} } \\ \begin{gathered} \ldots \hfill \\ I_{K} \hfill \\ \end{gathered} \\ \end{array} } \right]\;\;{\mathrm{with}}\quad {\mathbf{I}}(0) = \left[ {\begin{array}{*{20}c} {i_{0} } \\ 0 \\ 0 \\ \begin{gathered} \ldots \hfill \\ 0 \hfill \\ \end{gathered} \\ \end{array} } \right] $$

With the matrix elements:

5b $$ \left. {\begin{array}{*{20}l} {a_{m} = \beta_{m} - \gamma } \hfill \\ {b_{m + 1} = p\beta_{m} } \hfill \\ \end{array} } \right\}\quad m \ge 0 $$

The above system of equations can be solved recursively, starting with *I*_0_:

6 $$ \frac{{dI_{0} }}{dt} = + a_{0} I_{0} \Rightarrow I_{0} = i_{0} \exp [a_{0} t] $$

For *m* = 1, Eq. (5a) gives:

$$ \frac{{dI_{1} }}{dt} = + a_{1} I_{1} + b_{0} I_{0} $$

This equation can be solved, first by substituting Eq. (6) for *I*_0_, multiplying by exp(-*a*_1_*t*) and then integrating:

$$ I_{1} e^{{ - a_{1} t}} = b_{0} I_{0} \int\limits_{0}^{t} {\exp [(a_{0} - a_{1} )s]} \;ds $$

Noting that *a*_1_-*a*_0_ = *β*_*1*_ -*β*_*0*_ = Δ*β* and *b*_0_ = *pβ*_*0*_, we arrive at the solution:

$$ I_{1} = \frac{{p\beta_{0} }}{\Delta \beta }\left[ {e^{\Delta \beta t} - 1} \right]I_{0} (t) $$

This can then be used to find *I*_2_ and so on. In the “Appendix”, we show by induction that the infected population for a general variant, *I*_*m*_, is (for *m* > 0):

7 $$ I_{m} (t) = \left( {\frac{{p^{m} \beta_{m - 1} \ldots \beta_{1} \beta_{0} }}{{m!\Delta \beta^{m} }}} \right)\left( {{\mathrm{e}}^{\Delta \beta t} - 1} \right)^{m} I_{0} (t) $$

This equation can be rewritten as:

$$ I_{m} (t) = \frac{1}{m!}\left( {\frac{{\beta_{0} }}{\Delta \beta }} \right)^{(m)} \left[ {p\left( {{\mathrm{e}}^{\Delta \beta t} - 1} \right)} \right]^{m} I_{0} (t) $$

Note that *x*^(*m*)^ denotes the ascending factorial (Pochhammer’s function) of *x*. Thus, *J*_*K*_, the total infected population, up to order-*K*, is:

8 $$ J_{K} (t) = I_{0} (t) \cdot \sum\limits_{m = 0}^{K} {\frac{{(\beta_{0} /\Delta \beta )^{(m)} }}{m!}\left[ {p\left( {{\mathrm{e}}^{\Delta \beta t} - 1} \right)} \right]^{m} } $$

In general, this sum can be expressed in closed form using hyper-geometric functions. However, for the specific case of the limit as *K* → ∞, for *r* < 1, a simple closed form solution is possible. Using the notation *r* = *p*[exp(Δ*βt*)-1] and *x* = *β*_*0*_*/Δβ,* note that the ascending factorial satisfies:

$$ \frac{{x^{(m)} }}{m!} = \left( {\begin{array}{*{20}c} {x + m - 1} \\ m \\ \end{array} } \right) $$

Then, we can apply the generalized binomial theorem:

$$ J_{\infty } (t) = I_{0} (t) \cdot \sum\limits_{m = 0}^{\infty } {\left( {\begin{array}{*{20}c} {x + m - 1} \\ m \\ \end{array} } \right)r^{m} } = \frac{{I_{0} (t)}}{{(1 - r)^{x} }} $$

This result, in terms of the original symbols, is:

9 $$ J_{\infty } (t) = \frac{{I_{0} (t)}}{{\left[ {1 - p\left( {{\mathrm{e}}^{\Delta \beta t} - 1} \right)} \right]^{{\beta_{0} /\Delta \beta }} }} $$

This holds for *p*[exp(Δ*βt*)-1] < 1.

Equation (9) is the summation of a geometric series, with *r* = *p*[exp(Δ*βt*)-1]. Clearly, the ratio *r*, can be less than unity or greater than or equal to unity, depending on the value of *t*. This defines a critical value for *t*, namely:

10 $$ t_{c} = \frac{1}{\Delta \beta }\ln \left( {1 + \frac{1}{p}} \right) \approx \frac{1}{\Delta \beta }\ln \left( \frac{1}{p} \right) $$

When there are many variants (*K* → ∞), what does the trajectory look like? Let us consider this case and what is happening just before *t*_*c*_, namely *t* = *t*_*c*_*-δt*, with *δt* > 0 and *r* < 1, so:

11 $$ J_{\infty } (t) = \frac{{i_{0} \exp [a_{0} t]}}{{\left[ {1 - p\left( {{\mathrm{e}}^{\Delta \beta t} - 1} \right)} \right]^{{\beta_{0} /\Delta \beta }} }},\quad t < t_{c} $$

**Fig. 3:**
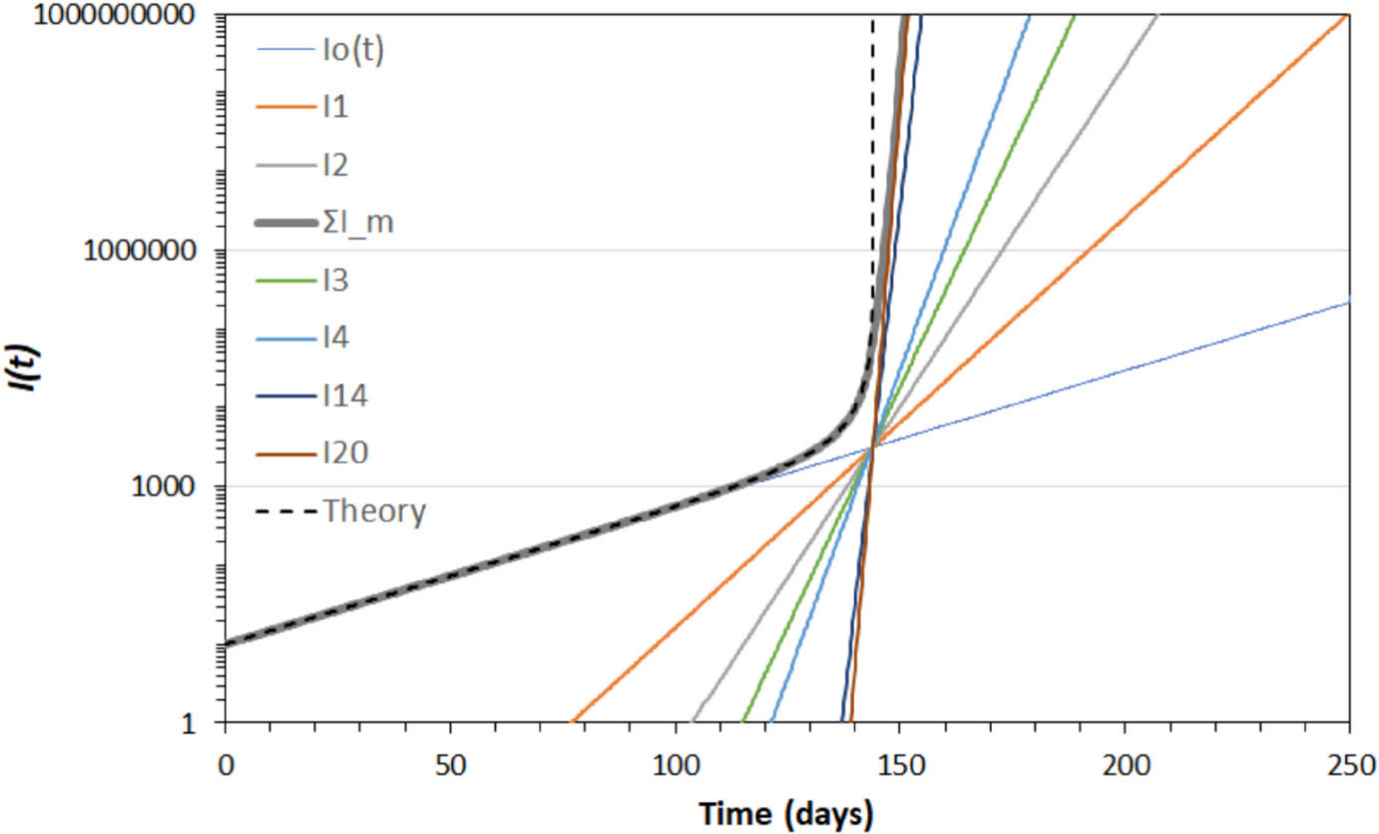
Explicit solutions of the rate Eqs. (4) and their exponential solutions for early stages, for several variants, given by Eq. (7). Also shown is the hyper-exponential growth of the total infected population, Eq. (8) for *K* = 20, and its limiting value, *J*_∞_, given by Eq. (9). The initial transmission rate is assumed to be *β* = 0.08 per day, increasing by Δ*β* = 0.08 with each successful mutation. The recovery time is 1/*γ* = 25 days. The probability of a successful mutation is *p* = 10^–5^ per infected individual. The initial values of the SIR parameters were: *I*_0_ = 10, S = 7 × 10^9^, *R* = 0. Based on these parameters, the critical time is *t*_*c*_≈144 days and at this time all variants have the same value *I*_*k*_(*t*_*c*_)≈3162

The growth described by Eqs. (7)–(9) is illustrated in Fig. 3. The total level of infection is seen to rise hyper-exponentially to a singularity at time *t*_*c*_, even though each variant by itself grows exponentially.

We can make use of some approximations (*K* → ∞, *t*_*c*_-*t* small) in Eq. (11) to show the asymptotic dependency and characterize the finite-time singularity. By considering what is happening just before *t*_*c*_, namely *t* = *t*_*c*_*-δt*, with *δt* > 0, so that *r* < 1, we get the power-law:

12 $$ J_{\infty } (t)\sim \left( {\frac{1}{{t_{c} - t}}} \right)^{{\frac{{\beta_{0} }}{\Delta \beta }}} $$

It is worth noting that the SEIR model of Halley et al. (2021) predicted a singularity exponent of unity, in contrast to the study of Schwarzendahl et al. that found an exponent of two (Schwarzendahl et al. 2022), while Eq. (12) shows that a wide range of exponents is possible in this model.

## Constrained hyper-exponential growth and collapse

In practice, the size of *I*(*t*) is limited by the size of the population, which is finite. Thus, hyper-exponential growth can proceed up to a point just before the critical time *t*_*c*_. Here, we can no longer assume that the epidemic is in a “early phase” of exponential growth (where *S*≈*N*). This can be approached by simulation. To obtain results from our model, following a standard approach and earlier papers (Brett and Rohani 2020; Carcione et al. 2020; Halley et al. 2021), we set up a series of difference equations to approximate the differential equations and solve it recursively, for each time-step, following the standard Euler approach. A virus with a transmission parameter *β*_0_ evolves into one with a parameter *β*_0_ + Δ*β* and an associated reproductive number *R*_0_^(1)^ (Order-1) according to the system of Eqs. (3). Further successful mutations (order-2, order-3 …) with transmission rates *β*_0_ + 2Δ*β*, *β*_0_ + 3Δ*β* and so on, lead to still higher reproductive numbers *R*_0_^(2)^, *R*_0_^(3)^ … respectively. We ignore strains that mutate to the same or lower *R*_0_, we do not include in the model complex mutations or “super-spreader” variants, and we do not make specific provisions for immunocompromised populations that may harbor viral persistence potentially initiating a novel variant. All calculations were performed in R (R Development Core Team 2023).

**Fig. 4:**
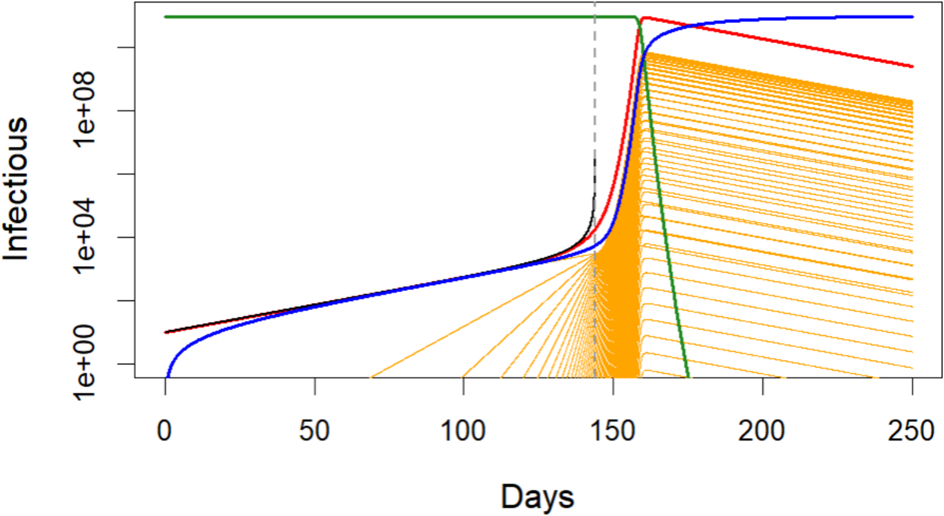
Numerical solution to Eqt. (3) with a finite population (*N* = 7 × 10^9^) for the first 100 strains. The red curve is the total number of infectious individuals (*J*), the green curve is the number of susceptibles (*S*) and the blue curve is the number of recovered (*R*) individuals. The orange curves are the numbers of infectious people with the different strains of the disease. The equations were solved using the Euler method with a step size Δ*t* = 0.1 day. The other parameters are the same as for Fig. 3. The black locus is the path of Eq. (9), showing the finite-time singularity expected with an infinite population size

In Fig. 4, we can see the epidemic growing initially at a rate of 0.04 per day, similar to the average growth of the COVID-19 pandemic (Halley et al. 2021). The first mutant strain with greater “fitness” arises at day 77, increasing more rapidly than the initial strain, and the second on day 104. There follows a rapid increase in the number of new variants because of the huge number of cases and susceptibles. Each new variant has a higher growth rate and thus comes to dominate the infectious population, leading to a hyper-exponential growth of the infectious population, where the slope is dominated by the latest variant. This process continues until the susceptible population is consumed by the epidemic to the extent that the number of susceptibles begins to fall significantly, which happens in Fig. 4 at around day 160. This leads to end of the cascade. After this, the infectious population also declines with a time-constant of recovery time. Subsequent new strains do not grow significantly. This results in a stabilization of the number of mutant strains. However, the number of susceptible individuals falls precipitously.

The probability of successful mutation, *p*, determines the output of this model. If the probability of successful mutation is very small, the system takes longer to reach the hyper-exponential stage and the hyper-exponential character is less pronounced. If it is large, there is a strong cascade effect with a marked curvature of the number of infectious individuals, implying sequential accelerations and HEG. For very small values (*p* < 10^–14^), by the time HEG kicks in, the epidemic has more or less finished and the overall patterns are virtually indistinguishable from typical SIR dynamics, despite new strains appearing. The curve diverges from the asymptotic result Eq. (12) because the maximum growth rate is limited by the finite population of susceptible individuals. In fact, Eq. (12) only strictly applies in the limit *N* → ∞.

In the rate equations, Eq. (4), the evolution of new strains is not limited by low density. According to Eq. (7), all strains are nonzero for any nonzero *I*_0_(*t*) and in Fig. 4, all strains begin to grow immediately. Suppose we define the “appearance” of a variant-*m* as its population being greater than unity. Then, with Eqs. (6) and (7), this requires:

13 $$ p^{m} \left( {{\mathrm{e}}^{\Delta \beta t} - 1} \right)^{m} i_{0} {\mathrm{e}}^{{a_{0} t}} \ge 1 $$

If we assume that this happens when exp(Δβt) ≫1, we have

$$ t_{m} \approx \frac{{m\Delta \beta t_{c} - \ln (i_{0} )}}{{m\Delta \beta + a_{0} }} $$

For example, using the parameters in Fig. 4, we require 77 days for the first variant and a similar calculation gives 104 days for the 2nd variant and for large values of *m* we have *t*_*m*_ → *t*_*c*_.

In the special case, where *i*_0_ = 1, we have:

14 $$ t_{m} \approx \frac{{t_{c} }}{{1 + \frac{{a_{0} }}{m\Delta \beta }}} $$

Note that all variants appear before *t*_*c*_.

An important question is when can an epidemic (or pandemic) take a hyper-exponential character. Clearly, this won’t happen if the epidemic starts to burn out before the arrival of the first variant. The time of the peak, *t*_*p*_, for SIR models has been calculated (Cadoni 2020; Turkyilmazoglu 2021). Thus, it is clearly necessary for a HEG character for

15 $$ t_{c} << t_{p} $$

## The stochastic character of the cascade process

The preceding calculation is approximate because, for any real strain, growth cannot begin until the strain actually appears with a population of unity. Let us consider Eq. (5b) for the first new strain.

$$ \frac{{dI_{1} }}{dt} = p\beta_{0} I_{0} $$

For very low values, the r.h.s. of this equation is better represented by a Poisson process with exponentially increasing rate *λ*(*t*) = *pβ*_0_*i*_0_.exp(*a*_0_*t*). The expected time of waiting to the first event for strain-1 is (Shih and Leemis 1993):

16 $$ T_{1} = \tau e^{q} E_{1} (q) $$

where *q* = *pβ*_0_*i*_0_/*a*_0_. In the example above, *p* = 10^–5^, *β*_0_ = 0.08, *a*_0_ = 0.04 and *I*_0_ = 10, so *q* = 20*p* = 2 × 10^–4^. Thus, we expect relatively small values for *q*. The exponential integral, *E*_1_(*q*), for *q* = 2 × 10^–4^ is about 7.94, so that *T*_1_ ~ 198.5, which is much greater than the waiting time under the deterministic calculation.

The same holds true for the other variants. Thus, the epidemic proceeds less rapidly to hyper-exponential behaviour and hence will be less often seen. The probability, *p*, of successful mutation, and the size of jumps to higher infectiousness Δ*β*, determine the move to HEG behaviour. The system is also limited by the size of the population, since HEG cannot happen if the epidemic burns out “too soon”. Clearly, the above equation for *T*_1_ leads to a sufficient criterion for *avoiding* HEG:

17 $$ t_{p} < \tau e^{q} E_{1} (q) $$

Contrary to concerns expressed earlier (Halley et al. 2021) HEG was not explicitly observed in the COVID-19 pandemic. This is expected because of active countermeasures taken, such as lockdowns and vaccinations. Instead, we observed the emergence and domination by a number of dominating variants.

In order to explore this issue, we ran a series of hybrid simulations of Eq. (3), following the method used earlier (Halley et al. 2021) for the SEIR model of SARS-CoV-2. In these, the final term in the differential equation for *I*_*m*_ is replaced by a Poisson random variable, δ_*m*-1_, namely:

18 $$ \frac{{dI_{m} }}{dt} = + (1 - p)\left( {\frac{{S\beta_{m} }}{N}} \right)I_{m} - \gamma I_{m} + \delta_{m - 1} ,\quad \forall 1 \le m \le K $$

**Fig. 5:**
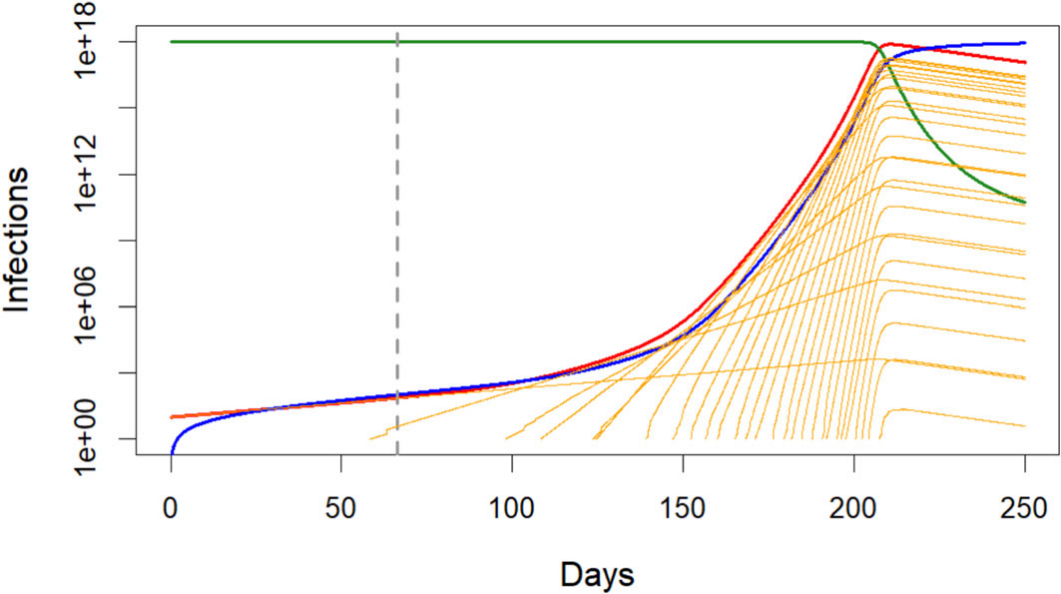
Numerical solution to Eq. (17) for the first 60 strains, with an initially large population of susceptibles (*N* = 10^18^), for one replicate simulation. The red curve is the total number of infectious individuals (*J*), the orange curves are the numbers of infectious with the different strains of the disease. The equations were solved using the Euler method with a step size Δ*t* = 0.05 days. The mutation probability is *p* = 5 × 10^–3^ and *γ* = 0.05, while other parameters are the same as in Fig. 4. The broken vertical line is the predicted position of the finite-time singularity in the absence of stochastic effects

Here the random variable δ_*m*-1_ is a Poisson process with a mean *p*(*S/N*)*β*_*m*-1_*I*_*m*-1_. Note that each realization of δ_*m*-1_ is non-unique, leading to a distribution of outcomes. Nevertheless, a single realization, such as we see in Fig. 5 below, is enough to illustrate certain points. The parameters used for Fig. 5, were different to those used in Figs. 3 and 4, with higher values of *p*, *β* and *N*. With the parameters of Fig. 4, as shown above, the first variant is expected to appear at *T*_1_ ~ 198.5, long after the peak. So, the epidemic is expected to burn out before the cascade develops. Here, as was found for the SEIR model of SARS-CoV-2, in this SIR model, the development of HEG is not in general quenched by stochasticity, though it is strongly delayed.

## Discussion and conclusions

The COVID-19 pandemic highlighted the ability of epidemics to evolve through the emergence of successive strains of greater infectiousness. It produced variants of SARS-CoV-2 with ever higher transmission potential, or a higher ability to infect previously low-risk groups. This prompted the insight that hyper-exponential growth (HEG) might arise in the growth of an epidemic (Halley et al. 2021; Schwarzendahl et al. 2022). Though pronounced HEG did not develop in the COVID pandemic, the model of Halley et al. (2021) showed that some features of COVID, such as the dominance of successive variants, could be predicted. Thus, the possibility of HEG is relevant in the consideration of future pandemics.

A major purpose of this study is to develop and study a real-world system, with known mechanistic properties, that gives rise to HEG. The concept of an epidemic model with evolving parameters of Halley et al. (2021) has been developed in greater depth and mathematical rigour. The model evolves a sequence of variants with successively higher infectiousness, each new strain growing faster and becoming the dominant strain, even as the other strains also grow exponentially. In this model, HEG develops directly from the evolving SIR process, rather than relying on any phenomenological dependence, such as the “*α*” parameter in Eq. (1). Thus, the eventual collapse of HEG, due to limited resources, can be seen in terms of basic mechanistic processes related to model parameters and initial conditions (see Fig. 4). Other important features of HEG can be expressed in terms of fundamental demographic parameters, such as the critical time to singularity, through Eq. (10), character of the singularity, Eq. (12), and necessary conditions for the emergence of HEG, through Eq. (15). This approach addresses more general questions, not only in epidemiology but also in wider applications.

The current study establishes the possibility of hyper-exponential growth within a specific mutation-structured SIR framework, still relatively preliminary in the context of more general mutation effects and realistic epidemic assumptions. The realisation and degree of HEG depends strongly on stochastic emergence. The HEG character is pronounced and becomes apparent only if the mutation probability is sufficiently high to allow the development of highly infectious strains before the pandemic burns out. This requires that the appearance time of new strains (Eq. (14)) and the critical time be smaller than the peak time, *t*_*p*_. The HEG phenomenon is delayed by the stochastic character of the host population. The rate Eqs. (4), having a solution given by Eq. (7), yield *I*_*m*_ > 0 and growing for all strains at any *t* > 0. In reality, new strains begin their growth when they actually appear, that is when their population is unity (*I*_*m*_ = 1). Thus, the expected waiting time, for any event, is greater than the waiting time under the deterministic calculation. So, in Fig. 5, we see that the epidemic proceeds less rapidly to hyper-exponential behaviour and hence will be less often seen in reality.

HEG is relatively rare in biology, perhaps because it often requires growth with the evolution of rates, and also because biological entities cannot persist in as state of HEG for very long. HEG is expected to drive any system rapidly to its limit causing a “phase change”, where other factors intervene. However, greater attention to this phenomenon may provide more observations. Another biological phenomenon, in which hyper-exponential growth has been inferred, is cancer. Typically, cancer begins from a neoplasm and then grows through uncontrolled mitosis. This has many of the features of an ecosystem (Frank and Nowak 2004; Merlo et al. 2006; Pienta et al. 2008; Attolini and Michor 2009). As cancerous growth proceeds, the cells or “clones” in the tumour shed all their developed features in the interest of greater growth. These clones compete with one another with the fastest-growing variant becoming the most abundant. Then another, faster, clone emerges and becomes dominant. For example, the fall of telomerase levels in tumours (Papadopoulou et al. 2003) can be seen as the transition from one type of cell to another. The connection between cancer and HEG has been discussed specifically by Hern who noted the similarity to human population growth (Hern 1993). Greater attention to the HEG phenomenon may yield better scientific and therapeutic dividends for cancer research and other areas of biology, especially those which involve evolution.

Modern human society exhibits HEG at several levels, with human population growth (prior to the demographic transition) being the best example. In technology, the current model for growth and development is Moore’s Law, where technology expands exponentially (Farmer and Lafond 2016). However, some technological growth appears to be hyper-exponential. Johansen & Sornette showed several measures of the global human economy to be growing hyper-exponentially (Johansen and Sornette 2001). In a recent review of the power consumption of data centers, median projections of usage suggested hyper-exponential growth: rising from 197.5 TWh (2010), to 299 TWh (2020) and up to 848 TWh (2030), though the ranges of uncertainty are large (Mytton and Ashtine 2022). HEG is to be expected from an economic model with baseline economic growth subject to constant innovations.

That the demographic transition of population growth occurred in a non-catastrophic manner is encouraging, but this does not mean that every complex system in a state of HEG will saturate in such a “gentle” fashion. In fact, a popular argument put forward by Diamond (Diamond 2011) is that many historical civilizations collapsed at, or close to, their apogee. Because numerous important phenomena in the modern world are expected to be HEG, it is imperative that we gain a better knowledge of this phenomenon. In addition to population and the size of the economy, we expect technology and knowledge generation to have a HEG character. For this reason, HEG will become increasingly important given the rapid transformations of our world. It is very important to build a solid theoretical understanding of HEG, especially in the presence of limited resources. In fact, it is surprising that so little work has been done on this theme.

## Electronic supplementary material

Below is the link to the electronic supplementary material.

Supplementary file1 (XLSX 738 kb)

Supplementary file2 (R 3 kb)

Supplementary file3 (R 4 kb)

## Data Availability

The R simulation code and XLSx spreadsheet that support the findings of this study are available within the paper’s Supplementary Information.

## Appendix

### A. Proof by induction of Eq. (7)

We see that all the solutions for the system of equations, Eq. (5), for *m* = 0 and *m* = 1, have the form:

$$ J_{m} (t) = \left( {\frac{{p^{m} \beta_{m - 1} \ldots \beta_{1} \beta_{0} }}{{m!\Delta \beta^{m} }}} \right)\left( {{\mathrm{e}}^{\Delta \beta t} - 1} \right)^{m} J_{0} (t) $$

Suppose it holds for a particular value, namely *m*.

Then:

$$ \frac{{dJ_{m + 1} }}{dt} - a_{m + 1} J_{m + 1} = b_{m} J_{m} = b_{m} \left( {\frac{{p^{m} \beta_{m - 1} \ldots \beta_{1} \beta_{0} }}{{m!\Delta \beta^{m} }}} \right)\left( {{\mathrm{e}}^{\Delta \beta t} - 1} \right)^{m} {\mathrm{e}}^{{a_{0} t}} I_{0} $$

In order to simplify things, introduce constants

$$ \lambda = \Delta \beta ,\quad A_{m} = b_{m} \frac{{p^{m} \beta_{m - 1} \ldots \beta_{1} \beta_{0} }}{{m!\Delta \beta^{m} }} $$

$$ \frac{d}{dt}\left[ {{\mathrm{e}}^{{ - a_{m + 1} t}} J_{m + 1} } \right] = A_{m} \left( {{\mathrm{e}}^{\lambda t} - 1} \right)^{m} {\mathrm{e}}^{{a_{0} t}} I_{0} {\mathrm{e}}^{{ - a_{m + 1} t}} $$

Use the binomial theorem: $$\left[ {{\mathrm{e}}^{\lambda t} - 1} \right]^{m} = \sum\limits_{k = 0}^{m} {\left( {\begin{array}{*{20}c} m \\ k \\ \end{array} } \right){\mathrm{e}}^{k\lambda t} \left( { - 1} \right)^{m - k} }$$.

Thus:

$$ \begin{aligned} \frac{d}{dt}\left[ {{\mathrm{e}}^{{ - a_{m + 1} t}} J_{m + 1} } \right] & = A_{m} I_{0} {\mathrm{e}}^{{a_{0} t - a_{m + 1} t}} \sum\limits_{k = 0}^{m} {\left( {\begin{array}{*{20}c} m \\ k \\ \end{array} } \right){\mathrm{e}}^{k\lambda t} \left( { - 1} \right)^{m - k} } \\ & = A_{m} I_{0} \sum\limits_{k = 0}^{m} {\left( {\begin{array}{*{20}c} m \\ k \\ \end{array} } \right)\left( { - 1} \right)^{m - k} {\mathrm{e}}^{{(k\lambda + a_{0} - a_{m + 1} )t}} } \\ \end{aligned} $$

Now we integrate:

$$ {\mathrm{e}}^{{ - a_{m + 1} t}} J_{m + 1} = A_{m} I_{0} \sum\limits_{k = 0}^{m} {\left( {\begin{array}{*{20}c} m \\ k \\ \end{array} } \right)\left( { - 1} \right)^{m - k} \int\limits_{0}^{t} {e^{{(k\lambda + a_{0} - a_{m + 1} )s}} ds} } $$

Note that *a*_m+1_-*a*_0_ = *β*_*m*+*2*_ -*β*_*0*_ = (*m* + 1)Δ*β* = (*m* + 1)λ, so

$$ {\mathrm{e}}^{{ - a_{m + 1} t}} J_{m + 1} = A_{m} I_{0} \sum\limits_{k = 0}^{m} {\left( {\begin{array}{*{20}c} m \\ k \\ \end{array} } \right)\left( { - 1} \right)^{m - k} \int\limits_{0}^{t} {e^{(k\lambda - (m + 1)\lambda )s} ds} } $$

and

$$ \begin{aligned} {\mathrm{e}}^{{ - a_{m + 1} t}} J_{m + 1} & = A_{m} I_{0} \sum\limits_{k = 0}^{m} {\left( {\begin{array}{*{20}c} m \\ k \\ \end{array} } \right)\left( { - 1} \right)^{m - k} \left. {\frac{{e^{(k - m - 1)\lambda s} }}{(k - m - 1)\lambda }} \right|}_{0}^{t} \\ & = A_{m} I_{0} \sum\limits_{k = 0}^{m} {\frac{m!}{{k!(m - k)!}}} \frac{{1 - e^{ - (m + 1 - k)\lambda t} }}{(m + 1 - k)\lambda }\left( { - 1} \right)^{m - k} \\ & = \frac{{A_{m} I_{0} }}{(m + 1)\lambda }\sum\limits_{k = 0}^{m} {\left( {\begin{array}{*{20}c} {m + 1} \\ k \\ \end{array} } \right)\left( {1 - e^{ - (m + 1 - k)\lambda t} } \right)} \left( { - 1} \right)^{m - k} \\ \end{aligned} $$

$$ \begin{aligned} {\mathrm{e}}^{{ - a_{m + 1} t}} J_{m + 1} & = ( - 1)\frac{{A_{m} I_{0} }}{(m + 1)\lambda }\sum\limits_{k = 0}^{m} {\left( {\begin{array}{*{20}c} {m + 1} \\ k \\ \end{array} } \right)} \left( { - 1} \right)^{m + 1 - k} (1)^{k} \\ &\quad + \frac{{A_{m} I_{0} }}{(m + 1)\lambda }\sum\limits_{k = 0}^{m} {\left( {\begin{array}{*{20}c} {m + 1} \\ k \\ \end{array} } \right)( + 1)^{k} \left( {e^{ - (m + 1 - k)\lambda t} } \right)} \left( { - 1} \right)^{m + 1 - k} \\ & = \frac{{A_{m} I_{0} }}{(m + 1)\lambda }\left( {1 - e^{ - \lambda t} } \right)^{m + 1} \\ \end{aligned} $$

And multiply both sides by exp(*a*_*m*+1_*t*):

$$ J_{m + 1} = \frac{{A_{m} I_{0} }}{(m + 1)\lambda }\left( {1 - e^{ - \Delta \beta t} } \right)^{m + 1} {\mathrm{e}}^{{a_{m + 1} t}} $$

Note that *a*_m+1_ = *a*_0_ + (*m* + 1)Δ*β*, so

$$ \begin{aligned} J_{m + 1} & = \frac{{A_{m} I_{0} }}{(m + 1)\Delta \beta }\left( {1 - e^{ - \Delta \beta t} } \right)^{m + 1} {\mathrm{e}}^{{a_{m + 1} t}} \\ & = \frac{{p^{m + 1} \beta_{m} \ldots \beta_{1} \beta_{0} }}{{(m + 1)!\Delta \beta^{m + 1} }}\left( {e^{\Delta \beta t} - 1} \right)^{m + 1} J_{0} (t) \\ \end{aligned} $$

Thus, we have:

$$ J_{m + 1} = \frac{{p^{m + 1} \beta_{m} \ldots \beta_{1} \beta_{0} }}{{(m + 1)!\Delta \beta^{m + 1} }}\left( {e^{\Delta \beta t} - 1} \right)^{m + 1} J_{0} (t) $$

Note that here we have used “$J_m$” to denote the number of infectious individuals of strain-m, whereas in the main text we denote this by “$I_m(t)$”.

### B. Waiting times in stochastic model

In a Poisson process with an exponentially increasing rate, the expected waiting time to the first event for is found as follows. The waiting time to the first event at time *t*, the probability that no event occurs on [0,*t*], is:

$$ P[X > t] = \exp \left[ { - \int\limits_{0}^{t} {\lambda (s)ds} } \right] $$

where the integral $\int\limits_{0}^{t} {\lambda (s)ds} = \int\limits_{0}^{t} {p\beta_{0} i_{0} \exp [a_{0} s]ds} = p\beta_{0} i_{0} \frac{{\exp [a_{0} t] - 1}}{{a_{0} }}$.

The mean of a random variable with CDF F(x) can be calculated:

$$ E[X] = \int\limits_{0}^{\infty } {xf(x)dx} = \int\limits_{0}^{\infty } {\int\limits_{0}^{x} {dt} f(x)dx} $$

Changing the order of integration yields (using Fubini’s theorem):

$$ E[X] = \int\limits_{0}^{\infty } {\int\limits_{t}^{\infty } {f(x)dx} } dt = \int\limits_{0}^{\infty } {\left[ {1 - F(t)} \right]} dt = \int\limits_{0}^{\infty } {P[X > t]} dt $$

Thus, the mean waiting time to the first event is:

$$ E[t_{1} ] = \int\limits_{0}^{\infty } {P[X > t]} dt = \int\limits_{0}^{\infty } {\exp \left[ { - \int\limits_{0}^{t} {\lambda (s)ds} } \right]} dt = \int\limits_{0}^{\infty } {\exp \left[ { - \frac{{p\beta_{0} i_{0} }}{{a_{0} }}\left( {\exp [a_{0} t] - 1} \right)} \right]} dt $$

The integral can be expressed in the form of an exponential integral E_1_(*x*) as follows:

$$ \int\limits_{0}^{\infty } {\exp \left[ { - \frac{{p\beta_{0} i_{0} }}{{a_{0} }}\left( {\exp [a_{0} t] - 1} \right)} \right]} dt = \int\limits_{0}^{\infty } {\exp \left[ { - q\left( {{\mathrm{e}}^{at} - 1} \right)} \right]} dt = e^{q} \int\limits_{0}^{\infty } {\exp \left[ { - q{\mathrm{e}}^{at} } \right]} dt $$

If we use a substitution of variables, *u* = *q*.exp(*at*), this becomes:

$$ e^{q} \int\limits_{0}^{\infty } {\exp \left[ { - q{\mathrm{e}}^{at} } \right]} dt = e^{q} \int\limits_{q}^{\infty } {\exp \left[ { - u} \right]} \frac{du}{{ua}} = \frac{{e^{q} }}{a}\int\limits_{q}^{\infty } {\frac{{\exp \left[ { - u} \right]}}{u}} du $$

Thus, the expected waiting time for strain-1 is:

$$ T_{1} = E[t_{1} ] = \frac{{e^{q} }}{{a_{0} }}E_{1} (q) = \tau e^{q} E_{1} (q) $$

where *q* = *pβ*_0_*i*_0_/*a*_0_. In the example above, *p* = 10^–5^, *β*_0_ = 0.08,* a*_0_ = 0.04 and *I*_0_ = 10, so *q* = 20*p* = 2 × 10^–4^. Thus, we expect relatively small values for *q*. The exponential integral for 2 × 10^–4^ is about 7.94, so that E_1_(t_1_) ~ 198.5, which is much greater than the waiting time under the deterministic calculation.

We can have an idea of this by using the inequality:

$$ \frac{1}{2}e^{ - q} \log \left( {1 + \frac{2}{q}} \right) \le E_{1} (q) \le e^{ - q} \log \left( {1 + \frac{1}{q}} \right) $$

Thus:

$$ \frac{\tau }{2}\log \left( {1 + \frac{2}{q}} \right) \le T_{1} \le \tau \log \left( {1 + \frac{1}{q}} \right) $$
